# Transfer accuracy of three different virtually designed CAD/CAM retainers – baseline results of a randomized clinical trial

**DOI:** 10.1007/s00784-026-06789-9

**Published:** 2026-03-09

**Authors:** Felix Linnerz, Rana Victoria Aghamiri, Inas Ayad, Mette Kuijpers, Jan Willem Hoekstra, Sachin Chhatwani, Gholamreza Danesh, Stephan Christian Möhlhenrich

**Affiliations:** 1 Department of Orthodontics, Witten/Herdecke University, Alfred-Herrhausen Str. 45, 58455 Witten, Germany; 2 Department of Dentistry, Section of Orthodontics, Dentofacial Orthopedics and Craniofacial Biology, Radboud University Medical Centre, THK 309, P.O. Box 9101, Nijmegen, 6500 HB The Netherlands

**Keywords:** Orthodontic retainers, Lingual retention, Computer-aided design, Transfer accuracy, Nickel–titanium, Cobalt–chromium

## Abstract

**Objectives:**

This study aimed to evaluate the immediate three-dimensional (3D) transfer accuracy of three virtually designed CAD/CAM lingual retainers fabricated from nickel–titanium (NiTi), titanium grade 5 (Ti5), and cobalt–chromium (CoCr). The investigation represents the baseline (T = 0) phase of a registered randomized clinical trial (DRKS00028974).

**Materials and methods:**

Sixty patients (32 females, 28 males; mean age 19.2 ± 6.9 years) were randomly allocated to receive one of the three CAD/CAM retainers. Immediately after bonding (T0–T1), intraoral scans were superimposed with the digital design to determine deviations at predefined interproximal contact points. Non-parametric Kruskal–Wallis tests with Dunn’s post-hoc comparisons were applied (p ≤ 0.05).

**Results:**

All 60 patients completed the baseline assessment. Transfer deviations were lowest in the NiTi group (0.17 mm, IQR 0.16–0.21), followed by Ti5 (0.37 mm, IQR 0.32–0.41) and CoCr (0.35 mm, IQR 0.30–0.40). NiTi showed significantly higher transfer accuracy compared with Ti5 (p < 0.001) and CoCr (p < 0.001). No significant difference was observed between Ti5 and CoCr (p > 0.999). No adverse events occurred.

**Conclusions:**

Laser-cut NiTi CAD/CAM retainers demonstrated significantly higher immediate transfer precision than milled Ti5 and CoCr retainers. These findings represent the baseline phase of the ongoing randomized clinical trial; subsequent analyses will determine whether such accuracy differences translate into clinically relevant stability outcomes.

**Clinical relevance:**

Accurate passive fit of CAD/CAM retainers is essential to prevent unwanted forces on teeth. Understanding material-dependent transfer deviations may improve digital bonding workflows and guide material selection in clinical orthodontics.

**Trial registration:**

DRKS00028974 (registered May 2022)

## Introduction

The retention phase is a crucial component of orthodontic treatment, designed to preserve functional efficiency and esthetic outcomes after active therapy. Relapse—the tendency of teeth to return to their pretreatment positions—has long been described as one of the most difficult problems in orthodontics. Long-term data confirm that mandibular anterior crowding may persist for decades, even in untreated normal occlusions [1]. These findings underline that relapse is a physiological process rather than a mere treatment failure, making retention indispensable.

Both removable and fixed retainers are widely used. Removable appliances are hygienic but depend heavily on patient compliance [2], Fixed lingual retainers, most commonly bonded on all anterior teeth from canine to canine, provide compliance-independent stability and are therefore widely used in the mandibular anterior region [3]. However, systematic reviews show no clear superiority among designs, leaving clinical practice guided mainly by individual preference and experience [4, 5].

Despite their advantages, fixed retainers present complications such as detachment, wire fractures, plaque accumulation, and unintended tooth movement [6, 7]. Torque differences (“X-effect”) and canine rotations (“twist-effect”) have also been reported, even with intact multistranded wires [6],, highlighting that precise passive fit and accurate transfer during bonding are crucial to prevent unwanted forces [8, 9].

Traditionally, lingual retainers were manually fabricated from multistranded stainless-steel wires on plaster models—an inexpensive but operator-dependent process prone to variability [7]. CAD/CAM workflows now enable standardized, reproducible retainers, often transferred with jigs for accurate bonding [10]. Clinical and laboratory studies have demonstrated that digitally designed retainers achieve high positioning accuracy and reproducibility [10, 11]. Wolf et al. showed that industrial custom-cute CAD/CAM retainers can achieve excellent fit and high accuracy [12], while randomized clinical trials confirmed improved periodontal parameters with CAD/CAM-NiTi retainers compared with conventional multistranded wires [13]. Koller et al. further confirmed high in vivo transfer precision when using customized CAD/CAM retainers with temporary adaptation threads [14].

Nickel–titanium retainers offer superelasticity and shape memory, titanium grade 5 exhibits high strength and biocompatibility, and cobalt–chromium provides durability and cost-efficiency [15]. Roser et al. showed that CAD/CAM-milled titanium and cobalt–chromium retainers achieved clinically acceptable adaptation, while in vitro comparisons reported that only titanium grade 5 matched the long-term mechanical performance of multistranded wires [15]. Alternative materials such as PEEK have been tested but remain limited to feasibility studies [16].

Although digital workflows offer promising accuracy and standardization, current evidence is still limited. Most trials compare CAD/CAM retainers with conventional stainless-steel wires [4, 5], while direct head-to-head comparisons between different CAD/CAM retainers are scarce [17]. Moreover, while survival rates and periodontal outcomes are frequently reported, the actual transfer accuracy of virtually planned retainers in vivo has been investigated only in few studies [10–12, 14]. Transfer precision is clinically critical, as even minor deviations during bonding may activate the retainer and induce unwanted forces [6].

Therefore, this baseline phase of a registered randomized clinical trial aimed to evaluate the immediate three-dimensional (3D) transfer accuracy of three digitally planned lingual retainers made of nickel–titanium, titanium grade 5, and cobalt–chromium, thereby assessing laboratory-fabricated CAD/CAM retainers relative to an established, commercially available industrial CAD/CAM reference system.

## Materials and methods

This study was designed as a parallel-group, randomized controlled clinical trial with a 1:1:1 allocation ratio. Participants were randomly assigned to one of three study groups (nickel–titanium, titanium grade 5, and cobalt–chromium retainers) using simple randomization, as described in the registered study protocol (DRKS00028974).

In accordance with the registered study protocol, group allocation followed a predefined simple randomization scheme based on a fair-coin procedure, implemented in two sequential steps to ensure equal allocation probability across the three study groups. The allocation sequence was generated and documented by an independent researcher not involved in any clinical procedures. Randomization was performed without stratification, as the study population was clinically homogeneous with respect to age, treatment indication, and retainer placement protocol across groups.

The present work represents an interim methodological analysis forming part of a larger randomized clinical trial registered in the German Clinical Trials Register (DRKS00028974), conducted at Witten/Herdecke University. While the registered trial primarily investigates long-term clinical outcomes, including Little’s Irregularity Index and periodontal parameters over a 12-month follow-up, the present manuscript reports a baseline methodological analysis, with transfer accuracy representing the primary outcome of this specific analysis rather than of the overall trial.

Accordingly, the present analysis is restricted to the baseline phase of the trial and evaluates the immediate three-dimensional (3D) transfer accuracy between the digitally planned retainer design (T0) and the clinically bonded retainer position (T1). Longitudinal results will be presented separately after completion of the one-year observation period. The overall trial received approval from the institutional ethics committee and was conducted and reported in accordance with the CONSORT 2025 statement for randomized clinical trials [18].

Due to the distinct handling characteristics of the materials, blinding of the operators was not feasible. Patients were informed about their participation in a comparative study but were not told the specific material assigned. The examiner conducting the 3D analysis was blinded to group allocation.

Retainers were bonded by trained clinicians under the supervision of an experienced orthodontist, following a standardized bonding protocol to ensure consistency. Measurement reliability was verified by repeating ten random superimpositions after one week, showing excellent intra-examiner agreement (ICC > 0.9).

A total of 60 patients (32 females, 28 males; mean age 19.2 ± 6.9 years) undergoing fixed appliance treatment were enrolled at the completion of their active orthodontic therapy. Baseline characteristics were comparable across the three groups. Table 1 summarizes the distribution of age and sex per group. Prior to participation, all individuals provided written informed consent. For underaged participants, consent was additionally secured from their parents or legal guardians. Owing to the immediate data collection, no exclusions due to follow-up loss were necessary.

**Table 1:** Baseline characteristics of the study participants by group

Characteristic	Group A (NiTi, *n* = 20)	Group B (Ti5, *n* = 20)	Group C (CoCr, *n* = 20)	Total (*n* = 60)
Age (years), mean ± SD	19.0 ± 5.8	18.7 ± 6.4	19.9 ± 8.5	19.2 ± 6.9
Sex (female/male)	10/10	12/8	10/10	32/28

Values are presented as mean ± standard deviation (SD) for continuous variables and as number (%) for categorical variables. Group A = NiTi, Group B = Ti5, Group C = CoCr

### Eligibility criteria

Inclusion criteria for the study are subjects with a need for fixed lingual fixed lingual retainers in the upper and lower jaw. The study included patients who had successfully completed comprehensive multibracket therapy with final alignment of the anterior teeth, demonstrated good oral hygiene as evaluated by the treating orthodontist, and exhibited intact anterior dentition from canine to canine in both jaws. Patients were excluded if they presented with active periodontal disease, restored or missing anterior teeth, craniofacial anomalies or syndromes, or showed poor compliance and an inability to attend scheduled follow-up appointments. All participants were recruited in our clinic between November 2022 and May 2025. No patient was excluded after randomization, and all completed the study protocol.

### Group allocation and retainer types

**Fig. 1:**
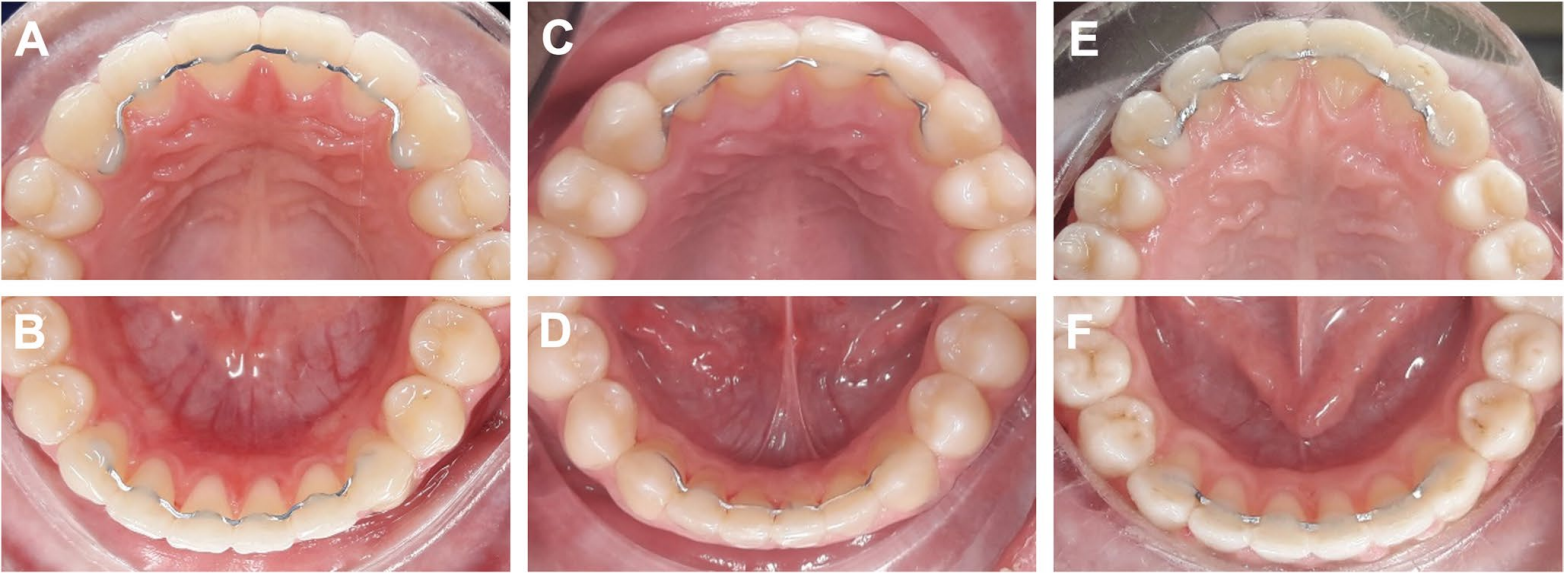
Clinical overview of the investigated retainer types: **A** + **B**: laser-cut nickel–titanium retainer, **C** + **D**: milled titanium Grade 5 retainer, and **E** + **F**: milled cobalt–chromium retainer

60 Participants were randomly assigned to one of three intervention groups. Each patient received the allocated type of digitally planned CAD/CAM lingual retainer in both the maxilla and mandible (Fig. 1):

- Group A: Laser-cut nickel–titanium retainer (Memotain^®^, CA Digital GmbH, Hilden, Germany).
- Group B: Milled titanium Grade 5 retainer (in-house production, University of Witten/Herdecke, Germany).
- Group C: Milled cobalt–chromium retainer (in-house production, University of Witten/Herdecke, Germany).

All retainers were designed for passive placement extending from canine to canine, adapted to the lingual surfaces at the level of the interproximal contact points. One retainer was placed in the maxilla and one in the mandible per patient, resulting in a total of 40 retainers per group for 20 patients.

### Digital workflow and retainer fabrication

Maximum 10 days prior to appliance removal, high-resolution intraoral scans (T0) of the maxilla and mandible were obtained using an iTero Element 2 scanner (Align Technology, San Jose, CA, USA).

In group A the Standard Tessellation Language (STL) files were transferred to the manufacturer (Memotain^®^, CA Digital GmbH, Hilden, Germany). The retainers were laser-cut from pre-shape-set NiTi sheets using high-precision industrial laser technology. The retainers were delivered pre-mounted on 3D-printed resin models with integrated transfer jigs for guided clinical placement.

In group B and C, the STL files were imported into the software OnyxCeph³™ (Image Instruments GmbH, Chemnitz, Germany), where the virtual design of the retainer was placed on the mid-lingual third of the anterior teeth. The design files were processed in the in-house Digital Competence Center, University of Witten/Herdecke. Retainers were milled from titanium Grade 5 (group B) or cobalt–chromium alloy blanks (group C) using a 5-axis milling unit (CORiTEC one+, imes-icore GmbH, Eiterfeld, Germany). After milling, each retainer was finished and pre-mounted on a stereolithographically printed transfer model with a custom positioning jig.

All CAD/CAM retainers across the three study groups were fabricated with identical dimensions, featuring a square cross-section of 0.4 × 0.4 mm. Identical design principles were applied across all groups in both software environments, including retainer extension from canine to canine, positioning at the interproximal contact level, and passive adaptation to the lingual tooth surfaces. All digitally planned retainers were clinically reviewed and approved for production by an experienced orthodontist (S.C.M.) prior to manufacturing.

### Clinical procedure

Enamel surfaces were etched using a 37% phosphoric acid gel (Orbis Ätzgel 37%, Orbis Dental, Germany). A universal bonding resin (Assure^®^ Universal Bonding Resin, Reliance Orthodontics, Itasca, IL, USA) was applied, and the retainers were bonded using a light-cured orthodontic composite according to a standardized protocol identical across all groups. Bonding procedures were performed by orthodontic residents of the department following a standardized, jig-guided protocol. Retainer positioning was clinically verified by experienced orthodontists (S.C./S.C.M.), with particular attention to gap-free adaptation at the canines. Initially, the retainers were bonded at the canines, followed by removal of the transfer jig and subsequent bonding of the remaining anterior teeth from one lateral incisor to the contralateral incisor. All transfer jigs across the three study groups were manufactured from the same type of material (Silagum-type silicone material). With regard to jig dimensions, the in-house fabricated transfer jigs (groups B and C) were designed based on the industrial reference jig (group A). Consequently, all transfer jigs had identical dimensions, extending to approximately the mid-point of the lateral incisors and overlapping the incisal edge by approximately 2 mm in the vestibular direction. A light-cured orthodontic adhesive (Transbond™, 3 M, St. Paul, MN, USA) was applied to the bonding pads of the retainers. Light curing was performed using a LED curing unit (miniLED^®^, Acteon/Satelec, France) with a nominal output of ≥ 1000 mW/cm². The light output was checked prior to clinical use according to the manufacturer’s specifications to ensure adequate irradiance.

### Transfer accuracy assessment

All 60 patients were included in the final analysis. Specifically, all 20 patients in each group (NiTi, Ti5, CoCr) were analyzed for the primary outcome. Outcome assessment was blinded; patients and treating clinicians were not blinded to the intervention. The primary outcome of the study was the 3D transfer accuracy of the retainers, expressed as deviations in millimeters between the digitally planned and the clinically achieved interproximal contact points (3 − 2, 2 − 1, 1–1) in both jaws. In addition, no secondary outcomes were prespecified as well as no long-term follow-up was conducted.

**Fig. 2:**
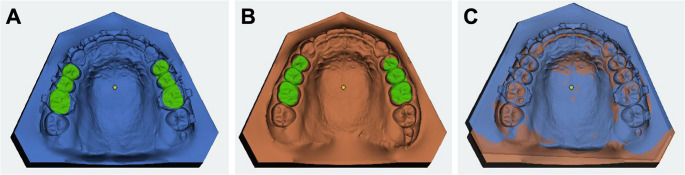
Visualization of the superimposition process using best-fit surface registration on the posterior teeth to avoid interference from the bonded retainers: intraoral scans before (**A**) and after (**B**) retainer bonding, as well as their superimposition (**C**)

During the same clinical appointment, a second intraoral scan (T1) was obtained after appliance removal and retainer bonding using the same iTero Element 2 device. The T0 and T1 datasets were superimposed in OnyxCeph³™ using the Onyx Inspect module (version 3.2; Image Instruments GmbH, Chemnitz, Germany). The 3D digital planning data of the custom-made NiTi retainers (group A) were provided by the manufacturer, whereas the corresponding STL datasets of the in-house planning (group B and C) were already available. Best-fit surface registration was that based on an iterative closest point (ICP) matching algorithm was performed on the posterior teeth to avoid positional bias from the bonded retainers, with the superimposition procedure and visualization of the 3D alignment process illustrated (Fig. 2).

**Fig. 3:**
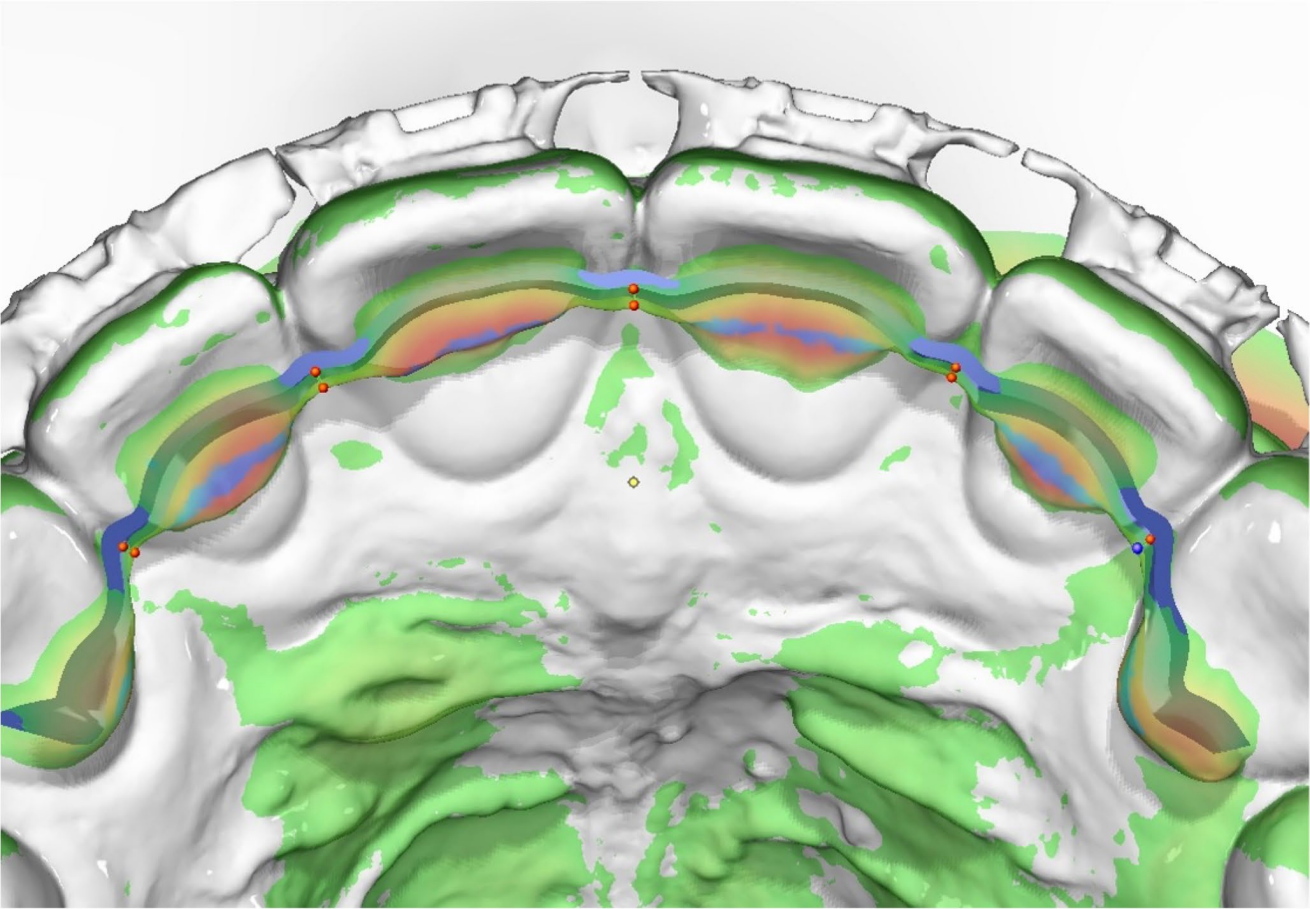
Deviation between the planned retainer design and its clinical position was assessed after superimposition, with red dots marking predefined interproximal measurement points at which the 3D linear distances between the digital design and the bonded retainer were calculated

For each arch, five predefined interproximal measurement points (3 − 2: canine–lateral, right and left site; 2 − 1: lateral–central, right and left site; 1–1: central–central) were identified in the T0 and T1 datasets and the shortest 3D linear distance between each corresponding point pair was calculated (Fig. 3).

### Sample size calculation

The sample size was calculated with G*Power software (Version 3.1.9.4 for Mac) based on the study results of Alrawas et al.[19], as no previous research had evaluated the transfer accuracy of retainers by superimposition. Assuming a standard deviation of 0.15 mm, a statistical power of 80%, and a Type I error of 0.05, a minimum of 32 retainers per group was required. To account for an estimated dropout rate of 25%, the final sample size was determined to be 40 retainers per group.

### Statistical analysis

Normality was assessed using the Shapiro–Wilk test, which indicated non-normal distribution of the continuous variables; therefore, non-parametric tests were applied. Continuous data are presented as median and interquartile range (IQR). To account for within-subject clustering arising from multiple measurement points within each arch, patient-level summary values were calculated as the mean deviation of the five measurement points per retainer (upper and lower jaw separately), and these summary values were used for all material comparisons. Although multiple measurement points were obtained per retainer, the effective unit of analysis was the individual retainer/patient. To account for non-independence of repeated measurements, transfer accuracy values were summarized per retainer before statistical comparison. The sample size calculation was therefore based on the number of retainers rather than on the total number of individual measurements. Group differences between materials were analyzed using the Kruskal–Wallis test with Bonferroni-adjusted Dunn’s post-hoc comparisons. Differences between the upper and lower jaw within each material were assessed using the Mann–Whitney U test. Interproximal comparisons (3–2, 2–1, 1–1) within each material and jaw were evaluated using the Kruskal–Wallis test with Bonferroni-corrected Dunn’s tests. All tests were two-sided with a significance level of *p* ≤ 0.05. No imputation was required, as all measurement points were available. All analyses were conducted using Prism (version 10, GraphPad Software Inc., La Jolla, CA, USA).

## Results

**Fig. 4:**
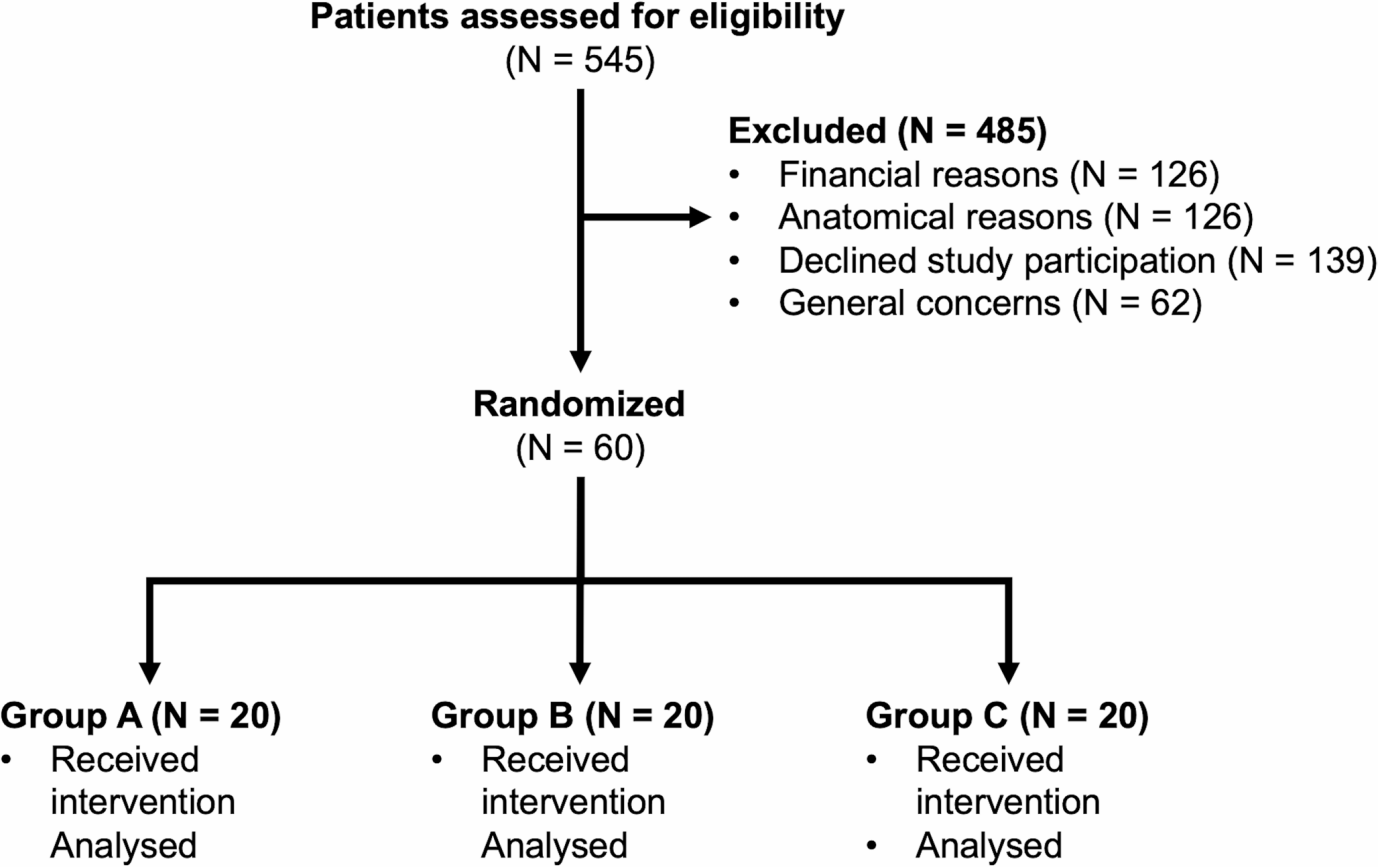
CONSORT flow diagram of patient recruitment and allocation. A total of 545 patients were assessed for eligibility, of whom 485 were excluded (158 for financial reasons, 126 due to anatomical limitations, 139 declined to participate, and 62 had general concerns regarding fixed retention). Sixty patients were randomized equally into three groups (*n* = 20 each). All patients received the allocated intervention and were included in the final analysis

A total of 545 patients were screened for eligibility, of whom 485 were excluded (158 for financial reasons, 126 for anatomical limitations, 139 declined participation, and 62 reported general concerns). Anatomical exclusion criteria included conditions preventing retainer adaptation or posing a risk of occlusal interference, such as pronounced lingual surface irregularities, abnormal crown morphology, or a persistent deep bite. Sixty patients were subsequently randomized into three equal groups (*n* = 20 per material). All participants received the allocated intervention and were included in the final analysis. The participant flow is summarized in the CONSORT diagram (Fig. 4).

No harms or adverse events occurred during the study. All retainer installations were performed exactly according to the study protocol, and no protocol deviations were reported. Consequently, no dropouts occurred, and all predefined measurement points could be assessed in both arches. This resulted in 200 measurements per material group, comprising 100 jaw-specific measurements (maxilla vs. mandible), 40 interproximal measurements in the regions 3–2 and 2–1, and 20 measurements in the region 1–1. Descriptive statistics for the pooled analyses are presented in Table 2, with a detailed breakdown by material, jaw, and interproximal contact point provided in Tables 3 and 4.

**Table 2:** Overall analysis by material group - Descriptive statistics of deviations (mm) for the three retainer materials (A = NiTi, B = Ti5, C = CoCr), pooled across all contact points and both jaws

Group	*N*	Median (IQR),	Mean	SD	Min	Max	95% CI
A	200	0.17 (0.16–0.21)	0.18	0.11	0	0.8	0.17–0.20
B	200	0.37 (0.32–0.41)	0.37	0.20	0	1	0.34–0.39
C	200	0.35 (0.30–0.40)	0.35	0.22	0	1.4	0.32–0.38

Values are presented as median and interquartile range (IQR), with mean, standard deviation (SD), minimum (Min), maximum (Max), and the 95% confidence interval (95% CI) of the mean provided for descriptive purposes. Each material group includes 200 measurements (20 patients × 2 jaws × 5 contact points)

**Table 3:** Analysis by material and jaw - Descriptive statistics of deviations (mm) per material group, separated by upper jaw (UJ) and lower jaw (LJ)

Material	Jaw	*N*		Median (IQR)	Mean	SD	Min	Max	95% CI
NiTi	Upper	100		0.18 (0.16–0.23)	0.20	0.11	0	0.7	0.17–0.22
	Lower	100		0.14 (0.14–0.21)	0.17	0.11	0	0.8	0.15–0.19
Ti5	Upper	100		0.45 (0.38–0.47)	0.43	0.19	0	1	0.39–0.46
	Lower	100		0.27 (0.24–0.38)	0.31	0.18	0	0.8	0.27–0.35
CoCr	Upper	100		0.35 (0.29–0.45)	0.37	0.24	0	1.4	0.32–0.41
	Lower	100		0.30 (0.25–0.40)	0.32	0.20	0	0.9	0.29–0.36

Displayed are the median and interquartile range (IQR) as primary descriptive measures, complemented by the mean, standard deviation (SD), minimum (Min), maximum (Max), and the 95% confidence interval (95% CI) of the mean for each material–jaw combination. Each jaw group includes 100 measurements (20 patients × 5 contact points)

**Table 4:** Analysis by material, jaw, and contact point - Descriptive statistics of deviations (mm) per material group, separated by upper and lower jaw, and interproximal contact point (ICP; 3 − 2, 2 − 1, 1–1)

Material	Jaw	ICP	*N*		Median (IQR)	Mean	SD	Min	Max	95% CI
NiTi	Upper	3 − 2	40		0.20 (0.13–0.42)	0.21	0.12	0.0	0.7	0.17–0.24
		2 − 1	40		0.20 (0.13–0.33)	0.18	0.10	0.0	0.4	0.15–0.21
		1–1	20		0.20 (0.16–0.34)	0.21	0.09	0.1	0.4	0.16–0.25
	Lower	3 − 2	40		0.20 (0.10–0.40)	0.18	0.14	0.0	0.8	0.13–0.22
		2 − 1	40		0.2 (0.12–0.32)	0.17	0.10	0.0	0.5	0.14–0.20
		1–1	20		0.15 (0.10–0.29)	0.16	0.08	0.0	0.3	0.12–0.20
Ti5	Upper	3 − 2	40		0.45 (0.35–0.67)	0.44	0.28	0.0	1.4	0.35–0.53
		2 − 1	40		0.30 (0.26–0.47)	0.32	0.20	0.0	1.0	0.25–0.38
		1–1	20		0.35 (0.26–0.46)	0.32	0.15	0.1	0.6	0.25–0.39
	Lower	3 − 2	40		0.3 (0.26–0.55)	0.34	0.20	0.1	0.8	0.28–0.41
		2 − 1	40		0.25 (0.23–0.44)	0.30	0.18	0.0	0.8	0.24–0.35
		1–1	20		0.30 (0.24–0.49)	0.34	0.22	0.1	0.9	0.24–0.44
CoCr	Upper	3 − 2	40		0.45 (0.41–0.71)	0.50	0.23	0.1	1.0	0.42–0.57
		2 − 1	40		0.40 (0.32–0.52)	0.38	0.17	0.0	0.8	0.32–0.43
		1–1	20		0.35 (0.33–0.55)	0.38	0.12	0.2	0.7	0.32–0.44
	Lower	3 − 2	40		0.30 (0.23–0.52)	0.32	0.19	0.0	0.8	0.26–0.38
		2 − 1	40		0.3 (0.23–0.43)	0.30	0.16	0.1	0.6	0.24–0.35
		1–1	20		0.20 (0.22–0.47)	0.32	0.21	0.1	0.8	0.22–0.41

Displayed are the median and interquartile range (IQR) as the primary descriptive measures, complemented by the mean, standard deviation (SD), minimum (Min), maximum (Max), and the 95% confidence interval (95% CI) of the mean for each material–jaw–interproximal-contact-point combination. Interproximal regions 3–2 and 2–1 include 40 measurements per group (20 patients × 2 sides), whereas region 1–1 includes 20 measurements per group (20 patients × 1 central contact point)

### Comparison between retainer materials

**Fig. 5:**
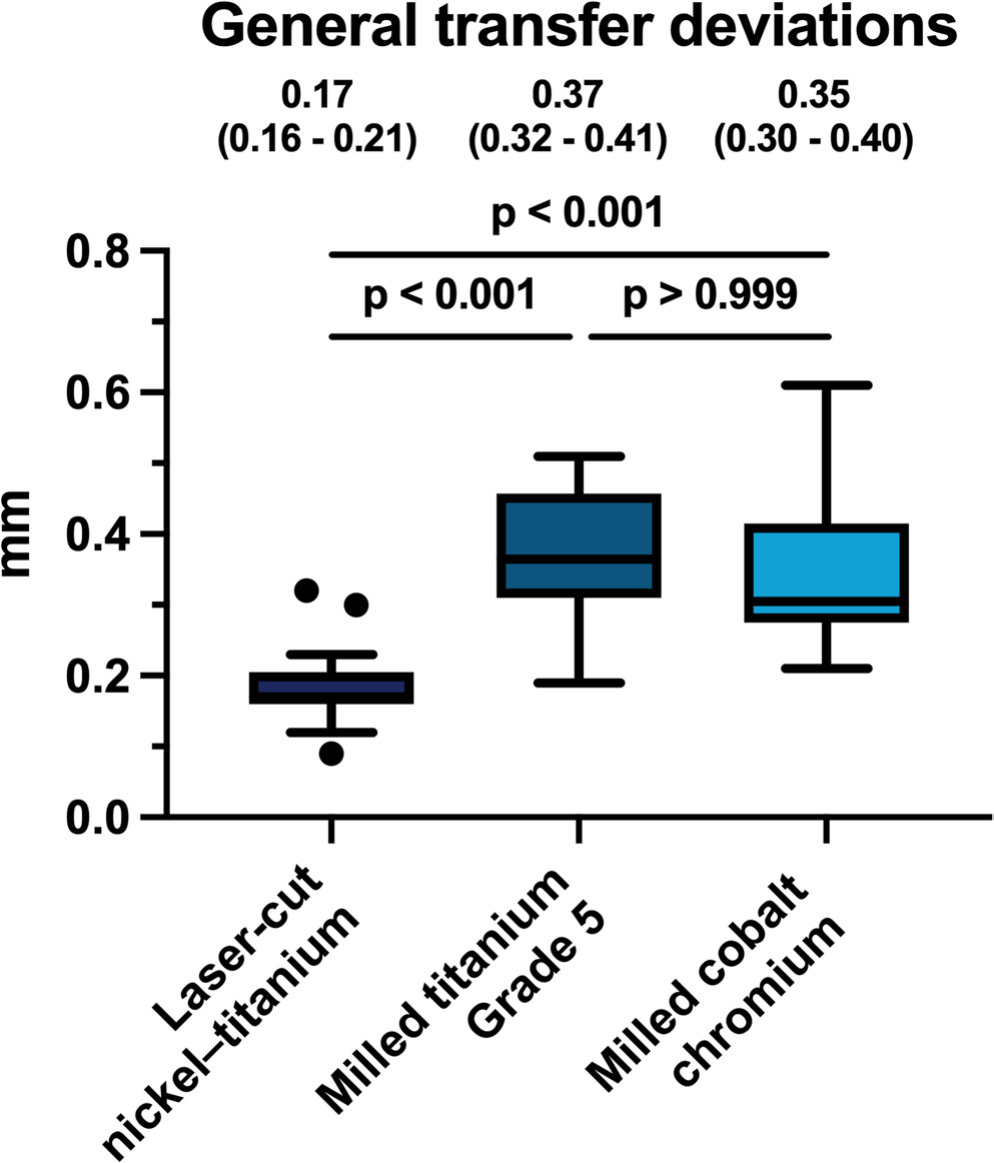
Tukey boxplots of overall 3D transfer deviations for the three CAD/CAM retainers (*N* = 20 per group). Data are shown as median and interquartile range (IQR). Group comparisons were performed using the Kruskal–Wallis test with Dunn’s post-hoc correction. Median (IQR) values are displayed above each box. Significance threshold: *p* < 0.05

Significant differences in transfer accuracy were observed between the three materials. Laser-cut NiTi retainers showed the lowest deviations with a median of 0.17 mm (IQR 0.16–0.21), which was significantly lower compared with milled titanium Grade 5 (0.37 mm, IQR 0.32–0.41; *p* < 0.001) and milled cobalt–chromium retainers (0.35 mm, IQR 0.30–0.40; *p* < 0.001). No significant difference was detected between the two milled retainers (*p* > 0.999). These findings indicate that the laser-cut NiTi retainers provided the most accurate transfer among the evaluated materials (Fig. 5).

### Arch-specific comparisons

**Fig. 6:**
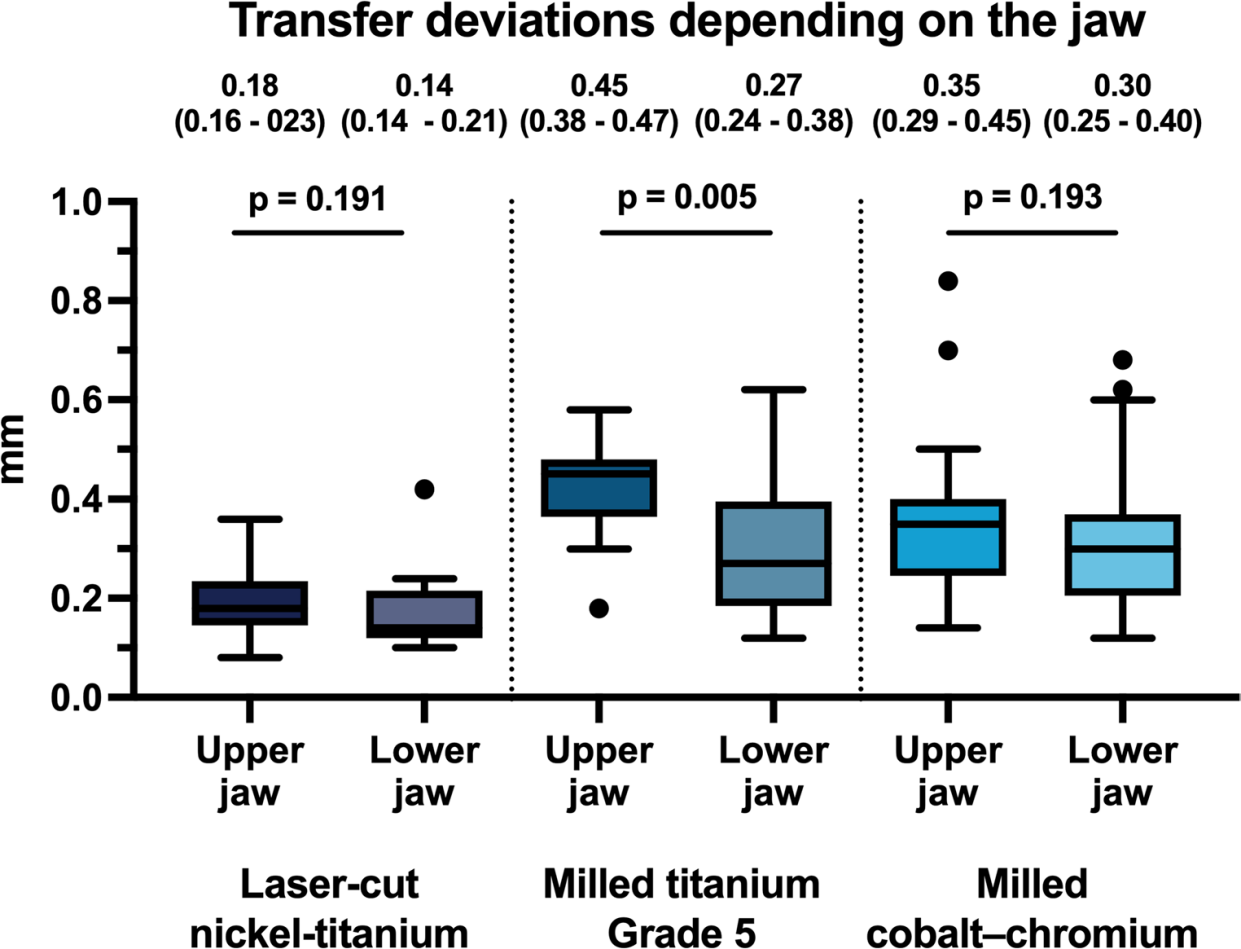
Bar chart illustrating the deviations in the transfer accuracy for the three different CAD/CAM-Retainers between the upper (UJ) and lower jaw (LJ) (each group: *N* = 100): **A**: Laser-cut nickel–titanium, **B**: Milled titanium Grade 5, **C**: Milled cobalt–chromium. Significance levels: * = *p* ≤ 0.05; ** = *p* ≤ 0.01; **** = *p* ≤ 0.001

When comparing the two arches, significant upper–lower differences were observed only for the milled titanium Grade 5 retainers, which showed greater deviations in the upper jaw (0.45 mm, IQR 0.38–0.47) than in the lower jaw (0.27 mm, IQR 0.24–0.38; *p* = 0.005). No corresponding differences were found for the laser-cut NiTi retainers (0.18 mm, IQR 0.16–0.23 vs. 0.14 mm, IQR 0.14–0.21; *p* = 0.191) or the milled cobalt–chromium retainers (0.35 mm, IQR 0.29–0.45 vs. 0.30 mm, IQR 0.25–0.40; *p* = 0.193), indicating comparable performance between the upper and lower jaw for these two materials (Fig. 6).

### Interproximal comparisons

**Fig. 7:**
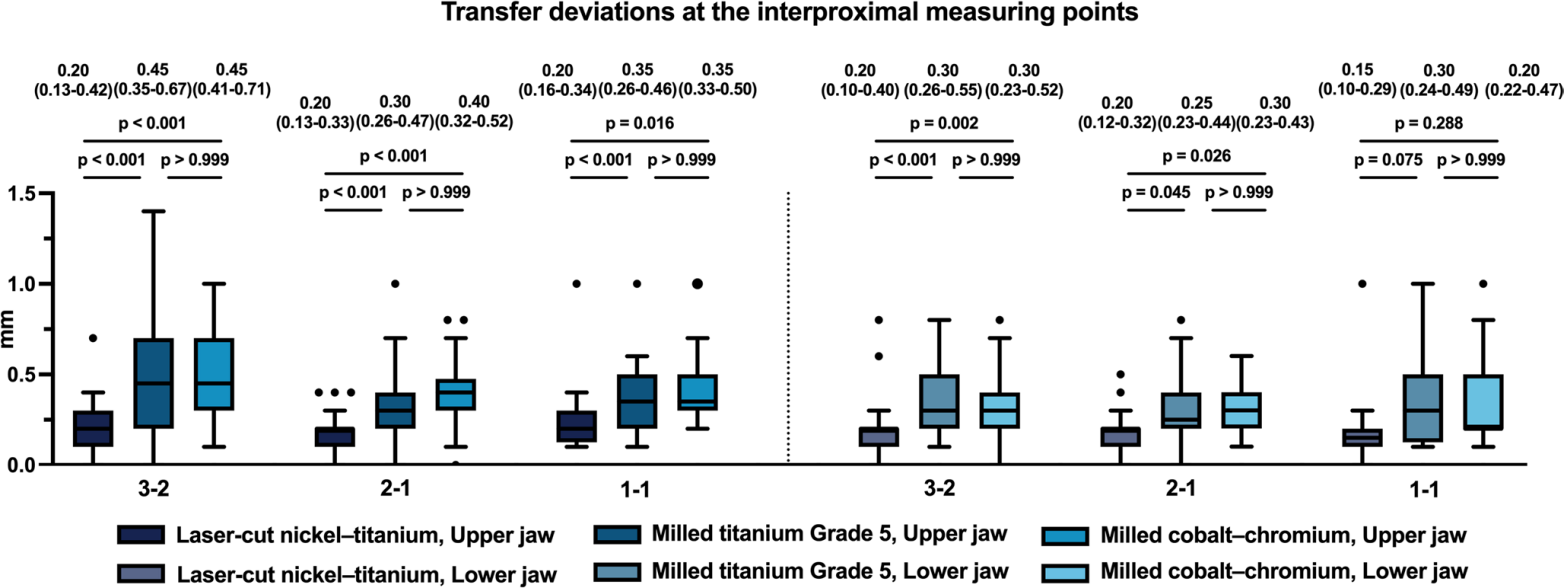
Bar chart illustrating the deviations in the transfer accuracy at the three different interproximal measurement regions (interproximal 3 − 2, 2 − 1: *N* = 40, interproximal 1–1: *N* = 20) between the three different CAD/CAM-Retainers: **A**: Laser-cut nickel–titanium, **B**: Milled titanium Grade 5, **C**: milled cobalt–chromium. Significance levels: * = *p* ≤ 0.05; ** = *p* ≤ 0.01; **** = *p* ≤ 0.001

**Fig. 8:**
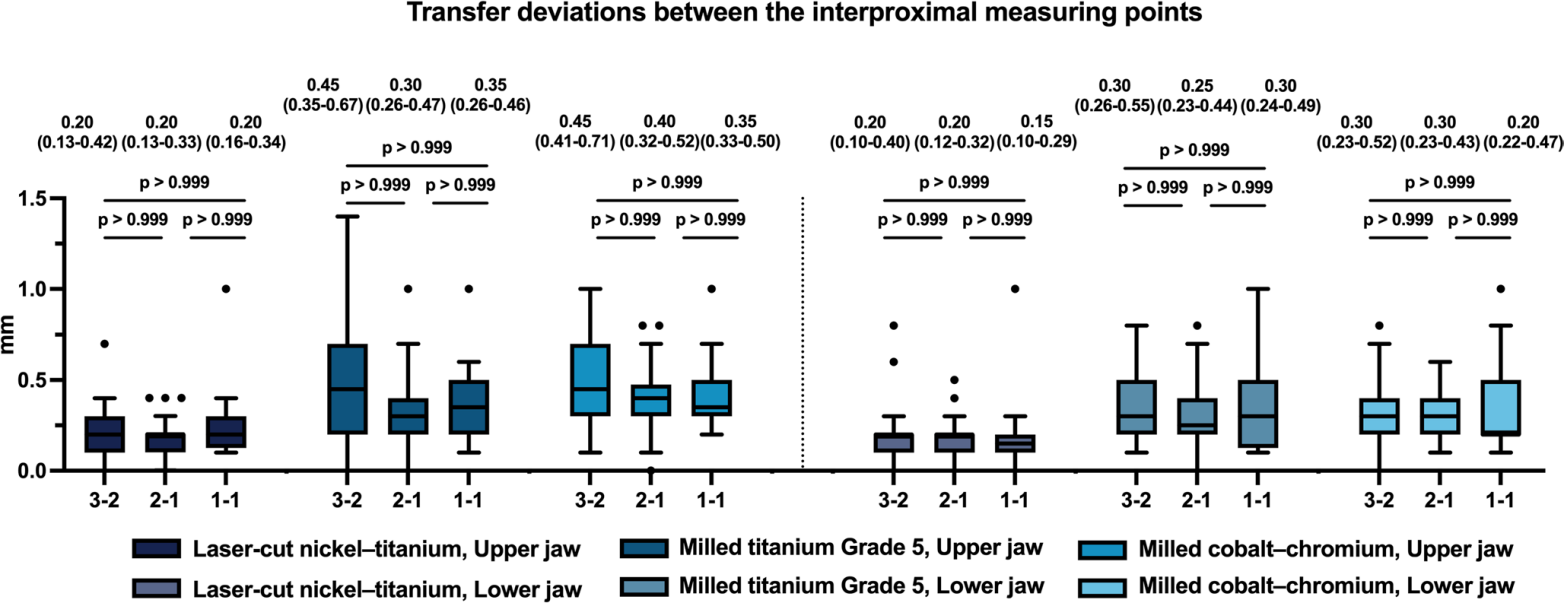
Bar chart illustrating the deviations in the transfer accuracy between the three different interproximal measurement regions (interproximal 3 − 2, 2 − 1: *N* = 40, interproximal 1–1: *N* = 20) for the three different CAD/CAM-Retainers: **A**: Laser-cut nickel–titanium, **B**: Milled titanium Grade 5, **C**: milled cobalt–chromium. Significance levels: * = *p* ≤ 0.05; ** = *p* ≤ 0.01; **** = *p* ≤ 0.001

A similar pattern emerged when examining the interproximal regions 3–2, 2–1, and 1–1 (Fig. 7). Across both arches, transfer deviations were significantly lower for the laser-cut NiTi retainers compared with both milled titanium Grade 5 and milled cobalt–chromium retainers (*p* < 0.001), except at the interproximal contact point 1–1, where no significant differences were detected (*p* ≥ 0.288). Within each material group, transfer deviations did not differ significantly among the interproximal regions (*p* > 0.999), suggesting consistent behaviour across measurement sites (Fig. 8).

## Discussion

The present randomized controlled clinical trial compared the transfer accuracy of three different virtual designed CAD/CAM retainers fabricated from laser-cut NiTi, Ti5, and CoCr. The main finding was that laser-cut NiTi retainers achieved the highest positional precision, while deviations in the Ti5 and CoCr groups were larger, especially in the vertical dimension and in the distal parts of the arch. These differences were not accompanied by clinical failures during the placement procedure, but they highlight material-dependent variations in intraoral accuracy that must be considered when choosing CAD/CAM retainers.

The different manufacturing workflows reflect current clinical CAD/CAM retention practice. The laser-cut NiTi retainer served as an established commercially available reference, whereas the Ti5 and CoCr retainers were fabricated using an in-house CAD/CAM workflow, allowing evaluation of laboratory-fabricated systems relative to an industrial standard and assessment of material-related differences. Nevertheless, workflow-related factors may have influenced transfer accuracy and should be considered when interpreting the results. In addition, differences in manufacturing workflows and fabrication techniques, such as laser cutting versus milling, may influence transfer accuracy due to variations in manufacturing tolerances, post-processing steps, and transfer jig characteristics. These workflow-related factors should therefore be considered when interpreting the observed differences.

Our findings align with earlier investigations on transfer precision. Wolf et al. first demonstrated that CAD/CAM-fabricated NiTi retainers can be positioned intraorally with deviations below 0.5 mm, with the majority of discrepancies occurring in the vertical axis [12]. More recent data from Koller et al. similarly revealed high overall accuracy for CAD/CAM titanium retainers, though significant vertical deviations increased towards the distal segments [14]. In a pilot study, Kang et al. compared computer-aided custom-cut and custom-bent retainers with conventional manual retainers and showed superior dimensional accuracy of custom-cut CAD/CAM retainers, whereas stability after six months of simulated aging did not differ between groups [10]. Cui et al. also confirmed high precision for CAD/CAM retainers manufactured by a bending machine, with only minor deviations along the y-axis for rectangular wires [11]. Together, these results and our trial indicate that CAD/CAM technology enables accurate transfer of retainers into the oral cavity, but vertical distortion and material properties must be considered.

From a clinical perspective, the accuracy of retainer transfer is crucial because even small deflections can transform a passive appliance into an active one, potentially inducing unintended tooth movements. Egli et al. reported that unexpected changes occurred in 17% of patients with directly bonded retainers, mostly involving lingual inclination of the mandibular canine [9]. Sifakakis et al. demonstrated in vitro that minimal deformations of 0.2 mm can generate forces exceeding 1 N and that residual forces persist even after unloading [20, 21]. Clinically, Wolf et al. reported that 56% of patients remained stable, while 30% showed moderate and 13% severe unintended movements, most often rotations of the anterior block [22]. Although the numerical difference in passive fit between groups in the present study was small, even minor discrepancies may be clinically relevant, as they can translate into measurable deflections and unwanted forces on the teeth [23]. Therefore, the more consistent passive fit observed in the NiTi CAD/CAM group may help minimize such effects and enhance long-term stability. It should be emphasized that the three-dimensional transfer accuracy assessed in this study represents a geometric surrogate outcome and does not directly assess force systems or long-term clinical effects. Accordingly, the clinical relevance of the observed differences should be interpreted with caution and will be addressed in the planned follow-up analyses of the registered trial.

Beyond accuracy, the long-term effectiveness of CAD/CAM retainers has been evaluated in several randomized controlled trials. Adanur-Atmaca et al. found that CAD/CAM NiTi retainers (Memotain) achieved comparable stability to multistranded stainless steel after one year, but gingival inflammation and calculus accumulation were lowest in the CAD/CAM group [13]. In this context, Alrawas et al. reported similar findings, with equivalent stability but significantly improved periodontal indices in the CAD/CAM group [19]. Elhosseiny et al. compared Ti5 CAD/CAM retainers with multistranded stainless steel and confirmed comparable maintenance of alignment over six months, while periodontal outcomes were significantly more favorable with CAD/CAM [24]. The multicenter RCT by Gera et al. demonstrated after six months no significant differences between CAD/CAM and conventional retainers regarding survival, stability, or patient satisfaction [25]. In a 24-month follow-up, Pullisaar et al. confirmed these findings, showing no differences in irregularity index or intercanine width between groups, while survival and cost outcomes were similar, with a slight advantage for CAD/CAM [26]. Collectively, these trials confirm that CAD/CAM retainers are at least as effective as conventional retainers for maintaining alignment in the short to medium term, with potential periodontal advantages.

These trial results have been synthesized in systematic reviews and meta-analyses. Bahije et al. concluded that fixed retention is more effective than removable retainers in the first six months, but evidence comparing different fixed systems was weak [3]. Al-Moghrabi et al. highlighted heterogeneity and low evidence quality, noting no clear superiority of any system [4]. The recent network meta-analysis by Bardideh et al. evaluated seven RCTs and found that CAD/CAM retainers may achieve slightly lower plaque indices and better short-term irregularity scores compared to some stainless steel wires, but overall stability and failure rates were similar [5]. Jedliński et al. reviewed causes of fixed retainer failures and concluded that no wire type guarantees stability, failures are common in the first six months, and fiber-reinforced composites are especially technique-sensitive [17]. Cochrane reviews consistently emphasized the lack of high-quality evidence favoring one retention method over another [27, 28]. These reviews reinforce the interpretation of our results: CAD/CAM retainers provide accuracy and hygiene advantages, but long-term superiority over conventional approaches remains unproven.

The material choice plays a central role in transfer accuracy and long-term behavior. Our findings of higher deviations in Ti5 and CoCr retainers are consistent with biomechanical studies. Roser et al. (A45) demonstrated that, under long-term artificial aging, Ti5 CAD/CAM retainers were the only digital type with failure rates and maximum load comparable to twistflex wires, while other CAD/CAM materials (NiTi sheets, CoCr, PEEK) showed higher failure rates and lower load capacity [15]. In this context, Möhlhenrich et al. reported that CAD/CAM NiTi retainers display superior elasticity and reduced permanent deformation compared to multistranded steel [29]. Arnold et al. and Baysal et al. showed that stainless steel wires differ markedly in torque resistance and deformation behavior, with five-strand wires being most resistant [6, 7], while Cooke and Sherriff demonstrated that rebonding significantly decreases bond strength [8]. This may contribute to failures in long-term use. Taken together, these biomechanical data suggest that while CAD/CAM retainers enable precise transfer, material selection critically affects durability and mechanical stability. Although not the primary focus of the present study, it should be acknowledged that rigid retainer materials, such as cobalt–chromium, may restrict physiologic tooth mobility. The potential long-term implications of such material-related effects on dental and periodontal health remain unclear and warrant further investigation. In addition, the clinical use of cobalt–chromium alloy is subject to ongoing scientific and regulatory discussion. Recent Medical Device Regulations and individual studies have raised considerations regarding cobalt–chromium dental alloys, while perspectives within the dental community remain heterogeneous [30].

Long-term clinical evidence, although limited, shows that fixed retention can be maintained for decades without adverse periodontal consequences. Booth et al. observed that after 20 years, 45 of 60 patients still had bonded mandibular retainers in situ, with only minimal irregularity and no detrimental effects on gingival health [1]. Renkema et al. as well as Segner and Heinrici similarly reported stable outcomes over 5 years, although early bonding failures were common [31–33]. These studies underline that survival of the retainer is more critical for long-term stability than the specific wire design. Our findings support this notion, as deviations in transfer accuracy did not translate into immediate failures, though their potential relevance may increase with longer observation. In this context, it should be noted that the retainer designs investigated in the present study were predominantly horizontally oriented and did not include vertical retainer extensions. Retainer designs incorporating vertical components may further influence transfer accuracy and should therefore be considered in future investigations.

The present study has limitations. The observation period was limited to the transfer process and immediate placement accuracy; long-term survival and biological effects were not evaluated. While comparisons were restricted to three CAD/CAM materials, other designs such as PEEK or fiber composites were not included. In addition, operator blinding was not feasible due to the visible differences in retainer materials and transfer jigs. Although standardized bonding protocols were applied and retainer positioning was verified by an experienced orthodontist, a potential influence of operator awareness on transfer accuracy cannot be completely excluded. Furthermore, the trial was conducted under controlled clinical conditions that may not fully reflect everyday practice. Nevertheless, the data contribute valuable evidence to the growing body of research on CAD/CAM retainers, particularly by directly comparing different digitally manufactured materials in a randomized setting. Finally, this study was conducted in a single university setting on young orthodontic patients. Therefore, the results may not directly generalize to older populations, different clinical environments, or long-term outcomes beyond the immediate transfer accuracy.

## Conclusion

In conclusion, laser-cut NiTi retainers achieved the highest transfer accuracy, while milled Ti5 and CoCr displayed greater deviations, especially in the vertical plane and distal regions. Previous clinical trials and systematic reviews confirm that CAD/CAM retainers are at least as effective as conventional multistranded retainers in maintaining stability, with possible advantages in periodontal health. However, long-term studies are required to confirm durability and to determine whether the superior transfer accuracy of NiTi translates into clinically meaningful benefits. For now, CAD/CAM retainers should be regarded as a promising alternative to conventional retainers, with material-specific properties guiding their clinical use.

## Data Availability

Data are available upon request from the corresponding author.

## References

[1] Booth FA, Edelman JM, Proffit WR (2008) Twenty-year follow-up of patients with permanently bonded mandibular canine-to-canine retainers. Am J Orthod Dentofacial Orthop 133:70–6. 10.1016/j.ajodo.2006.10.023 18174074 10.1016/j.ajodo.2006.10.023

[2] Forde K, Storey M, Littlewood SJ, Scott P, Luther F, Kang J (2018) Bonded versus vacuum-formed retainers: a randomized controlled trial. Part 1: stability, retainer survival, and patient satisfaction outcomes after 12 months. Eur J Orthod 40:387–398. 10.1093/ejo/cjx058 29059289 10.1093/ejo/cjx058

[3] Bahije L, Ennaji A, Benyahia H, Zaoui F (2018) A systematic review of orthodontic retention systems: the verdict. Int Orthod 16:409–424. 10.1016/j.ortho.2018.06.023 30001980 10.1016/j.ortho.2018.06.023

[4] Al-Moghrabi D, Pandis N, Fleming PS (2016) The effects of fixed and removable orthodontic retainers: a systematic review. Prog Orthod 17:24. 10.1186/s40510-016-0137-x 27459974 10.1186/s40510-016-0137-x PMC4961661

[5] Bardideh E, Ghorbani M, Shafaee H, Saeedi P, Younessian F (2023) A comparison of CAD/CAM-based fixed retainers versus conventional fixed retainers in orthodontic patients: a systematic review and network meta-analysis. Eur J Orthod 45:545–557. 10.1093/ejo/cjad033 37471113 10.1093/ejo/cjad033

[6] Arnold DT, Dalstra M, Verna C (2016) Torque resistance of different stainless steel wires commonly used for fixed retainers in orthodontics. J Orthod 43:121–9. 10.1080/14653125.2016.1155814 27104351 10.1080/14653125.2016.1155814

[7] Baysal A, Uysal T, Gul N, Alan MB, Ramoglu SI (2012) Comparison of three different orthodontic wires for bonded lingual retainer fabrication. Korean J Orthod 42:39–46. 10.4041/kjod.2012.42.1.39 23112930 10.4041/kjod.2012.42.1.39 PMC3481967

[8] Cooke ME, Sherriff M (2010) Debonding force and deformation of two multi-stranded lingual retainer wires bonded to incisor enamel: an in vitro study. Eur J Orthod 32:741–6. 10.1093/ejo/cjq017 20547494 10.1093/ejo/cjq017

[9] Egli F, Bovali E, Kiliaridis S, Cornelis MA (2017) Indirect vs direct bonding of mandibular fixed retainers in orthodontic patients: comparison of retainer failures and posttreatment stability. A 2-year follow-up of a single-center randomized controlled trial. Am J Orthod Dentofacial Orthop 151:15–27. 10.1016/j.ajodo.2016.09.009 28024770 10.1016/j.ajodo.2016.09.009

[10] Kang SH, Kwon JS, Chung CJ, Cha JY, Lee KJ (2022) Accuracy and stability of computer-aided customized lingual fixed retainer: a pilot study. Prog Orthod 23:39. 10.1186/s40510-022-00436-1 36404385 10.1186/s40510-022-00436-1 PMC9676156

[11] Cui FP, Park JJ, Kim SH (2024) Accuracy of lingual fixed retainers fabricated using a CAD/CAM bending machine. Korean J Orthod 54:257–263. 10.4041/kjod24.078 39048921 10.4041/kjod24.078 PMC11270144

[12] Wolf M, Schumacher P, Jager F, Wego J, Fritz U, Korbmacher-Steiner H, Jager A, Schauseil M (2015) Novel lingual retainer created using CAD/CAM technology: evaluation of its positioning accuracy. J Orofac Orthop 76:164–74. 10.1007/s00056-014-0279-8 25744094 10.1007/s00056-014-0279-8

[13] Adanur-Atmaca R, Cokakoglu S, Ozturk F (2021) Effects of different lingual retainers on periodontal health and stability. Angle Orthod 91:468–476. 10.2319/110220-904.1 33587126 10.2319/110220-904.1 PMC8259753

[14] Koller S, Craveiro RB, Niederau C, Pollak TL, Knaup I, Wolf M (2023) Evaluation of digital construction, production and intraoral position accuracy of novel 3D CAD/CAM titanium retainers. J Orofac Orthop 84:384–391. 10.1007/s00056-022-00393-8 35357509 10.1007/s00056-022-00393-8 PMC10587025

[15] Roser CJ, Bauer C, Hodecker L, Zenthofer A, Lux CJ, Rues S (2025) Comparison of six different CAD/CAM retainers vs. the stainless steel twistflex retainer: an in vitro investigation of survival rate and stability. J Orofac Orthop 86:119–128. 10.1007/s00056-023-00486-y 37378840 10.1007/s00056-023-00486-y PMC11861246

[16] Aboulazm K, von See C, Othman A (2021) Fixed lingual orthodontic retainer with bilateral missing lateral incisors produced in PEEK material using CAD/CAM technology. J Clin Exp Dent 13:e549–e551. 10.4317/jced.58035 34188759 10.4317/jced.58035 PMC8223158

[17] Jedlinski M, Grocholewicz K, Mazur M, Janiszewska-Olszowska J (2021) What causes failure of fixed orthodontic retention? - systematic review and meta-analysis of clinical studies. Head Face Med 17:32. 10.1186/s13005-021-00281-3 34301280 10.1186/s13005-021-00281-3 PMC8306281

[18] Hopewell S, Chan AW, Collins GS, Hrobjartsson A, Moher D, Schulz KF, Tunn R, Aggarwal R, Berkwits M, Berlin JA, Bhandari N, Butcher NJ, Campbell MK, Chidebe RCW, Elbourne D, Farmer A, Fergusson DA, Golub RM, Goodman SN, Hoffmann TC, Ioannidis JPA, Kahan BC, Knowles RL, Lamb SE, Lewis S, Loder E, Offringa M, Ravaud P, Richards DP, Rockhold FW, Schriger DL, Siegfried NL, Staniszewska S, Taylor RS, Thabane L, Torgerson D, Vohra S, White IR, Boutron I (2025) CONSORT 2025 statement: updated guideline for reporting randomised trials. BMJ 389:e081123. 10.1136/bmj-2024-081123 40228833 10.1136/bmj-2024-081123 PMC11995449

[19] Alrawas MB, Kashoura Y, Tosun O, Oz U (2021) Comparing the effects of CAD/CAM nickel-titanium lingual retainers on teeth stability and periodontal health with conventional fixed and removable retainers: a randomized clinical trial. Orthod Craniofac Res 24:241–250. 10.1111/ocr.12425 32865325 10.1111/ocr.12425

[20] Sifakakis I, Eliades T, Bourauel C (2015) Residual stress analysis of fixed retainer wires after in vitro loading: can mastication-induced stresses produce an unfavorable effect? Biomed Tech (Berl) 60:617–22. 10.1515/bmt-2015-0013 26057215 10.1515/bmt-2015-0013

[21] Sifakakis I, Pandis N, Eliades T, Makou M, Katsaros C, Bourauel C (2011) In-vitro assessment of the forces generated by lingual fixed retainers. Am J Orthod Dentofacial Orthop 139:44–8. 10.1016/j.ajodo.2010.02.029 21195275 10.1016/j.ajodo.2010.02.029

[22] Wolf M, Schulte U, Kupper K, Bourauel C, Keilig L, Papageorgiou SN, Dirk C, Kirschneck C, Daratsianos N, Jager A (2016) Post-treatment changes in permanent retention. J Orofac Orthop 77:446–453. 10.1007/s00056-016-0054-0 27761588 10.1007/s00056-016-0054-0

[23] Shaughnessy TG, Proffit WR, Samara SA (2016) Inadvertent tooth movement with fixed lingual retainers. Am J Orthod Dentofacial Orthop 149:277–86. 10.1016/j.ajodo.2015.10.015 26827985 10.1016/j.ajodo.2015.10.015

[24] Elhosseiny NEA, Marzouk WM, Tageldin MA (2025) Effectiveness of CAD/CAM titanium fixed lingual retainer versus conventional stainless steel fixed retainer (randomized controlled clinical trial). Clin Oral Investig 29:343. 10.1007/s00784-025-06418-x 40528130 10.1007/s00784-025-06418-x PMC12174219

[25] Gera A, Pullisaar H, Cattaneo PM, Gera S, Vandevska-Radunovic V, Cornelis MA (2023) Stability, survival, and patient satisfaction with CAD/CAM versus conventional multistranded fixed retainers in orthodontic patients: a 6-month follow-up of a two-centre randomized controlled clinical trial. Eur J Orthod 45:58–67. 10.1093/ejo/cjac042 35964235 10.1093/ejo/cjac042 PMC9912708

[26] 26. Pullisaar H, Cattaneo PM, Gera A, Sankiewicz M, Bilinska M, Vandevska-Radunovic V and Cornelis MA (2024) Stability, survival, patient satisfaction, and cost-minimization of CAD/CAM versus conventional multistranded fixed retainers in orthodontic patients: a 2-year follow-up of a two-centre randomized controlled trial. Eur J Orthod 46. doi: 10.1093/ejo/cjae006 10.1093/ejo/cjae006 PMC10888518 38394353

[27] Littlewood SJ, Millett DT, Doubleday B, Bearn DR, Worthington HV (2016) Retention procedures for stabilising tooth position after treatment with orthodontic braces. Cochrane Database Syst Rev 2016:CD002283. 10.1002/14651858.CD002283.pub4 26824885 10.1002/14651858.CD002283.pub4 PMC7138206

[28] Martin C, Littlewood SJ, Millett DT, Doubleday B, Bearn D, Worthington HV, Limones A (2023) Retention procedures for stabilising tooth position after treatment with orthodontic braces. Cochrane Database Syst Rev (5):CD002283. 10.1002/14651858.CD002283.pub5 10.1002/14651858.CD002283.pub5 PMC10202160 37219527

[29] Möhlhenrich SC, Jager F, Jager A, Schumacher P, Wolf M, Fritz U, Bourauel C (2018) Biomechanical properties of CAD/CAM-individualized nickel-titanium lingual retainers: an in vitro study. J Orofac Orthop 79:309–319. 10.1007/s00056-018-0144-2 30014179 10.1007/s00056-018-0144-2

[30] Vaicelyte A, Janssen C, Le Borgne M, Grosgogeat B (2020) Cobalt–Chromium dental alloys: metal exposures, toxicological risks, CMR classification, and EU regulatory framework. Crystals 10:1151. 10.3390/cryst10121151

[31] Renkema AM, Al-Assad S, Bronkhorst E, Weindel S, Katsaros C, Lisson JA (2008) Effectiveness of lingual retainers bonded to the canines in preventing mandibular incisor relapse. Am J Orthod Dentofacial Orthop 134:179e1-8. 10.1016/j.ajodo.2008.06.003 18675196 10.1016/j.ajodo.2008.06.003

[32] Renkema AM, Renkema A, Bronkhorst E, Katsaros C (2011) Long-term effectiveness of canine-to-canine bonded flexible spiral wire lingual retainers. Am J Orthod Dentofacial Orthop 139:614–21. 10.1016/j.ajodo.2009.06.041 21536204 10.1016/j.ajodo.2009.06.041

[33] Segner D, Heinrici B (2000) Bonded retainers–clinical reliability. J Orofac Orthop 61:352–8. 10.1007/pl00001905 11037687 10.1007/pl00001905

